# Erratum to “The Basic Cardiovascular Responses to Postural Changes, Exercise, and Cold Pressor Test: Do They Vary in Accordance with the Dual Constitutional Types of Ayurveda?”

**DOI:** 10.1155/2011/328380

**Published:** 2011-01-18

**Authors:** Piyush Kumar Tripathi, Kishor Patwardhan, Girish Singh

**Affiliations:** ^1^Department of Kriya Sharir, Faculty of Ayurveda, Institute of Medical Sciences, Banaras Hindu University, Varanasi, Uttar Pradesh 221005, India; ^2^Division of Biostatistics, Department of Community Medicine, Institute of Medical Sciences, Banaras Hindu University, Varanasi, Uttar Pradesh 221005, India

The authors would like to apologise for the following errors that the original paper contains [1].

## 1. Last Sentence in the Abstract

*Present version:* However, we noted a significant fall in the diastolic blood pressure immediately after performing the isotonic exercise for five minutes, in *Vata-Kapha *individuals in comparison to the other two groups, namely, *Pitta-Kapha *and *Vata-Pitta*.

*To be corrected as:* However, we noted that the rise in diastolic blood pressure recorded immediately after performing the isotonic exercise for five minutes was significantly minimal in *Vata-Kapha *individuals in comparison to the other two groups, namely, *Pitta-Kapha *and *Vata-Pitta*.

## 2. Last Sentence in the Observations Section

*Present version:* This means that the fall in DBP was significant in VK group in comparison to VP and PK groups.

*To be corrected as:* This means that the rise in DBP was significantly minimal in VK group in comparison to VP and PK groups.

## 3. First Sentence in the Last Paragraph of the Discussion Section

*Present version:* A significant fall in DBP among *Vata-Kapha *individuals, recorded immediately after performing the isotonic exercise for five minutes, in comparison to *Pitta-Kapha *and *Vata-Pitta *individuals is another observation that is noteworthy.

*To be corrected as:* A significantly minimal increase in DBP among *Vata-Kapha *individuals, recorded immediately after performing the isotonic exercise for five minutes, in comparison to *Pitta-Kapha *and *Vata-Pitta *individuals is another observation that is noteworthy.

## 4. Fifth Sentence in the Last Paragraph of the Discussion Section

*Present version:* As the study suggests, vasodilatation and resultant fall in peripheral resistance predominates among *Vata-Kapha* individuals after the isotonic exercise in comparison to the other two groups.

*To be corrected as:* As the study suggests, vasodilatation predominates among *Vata-Kapha* individuals after the isotonic exercise in comparison to the other two groups.

## 5. Second Sentence in the Summary and Conclusion Section

*Present version:* Though, the study indicates that the basic cardiovascular responses do not significantly vary in accordance with the dual constitutional types of Ayurveda, it suggests that the fall in DBP recorded immediately after performing the isotonic exercise for five minutes varies significantly in relation to *Prakriti *groups and this fall is significant among VK group in comparison to VP and PK groups.

*To be corrected as:* Though the study indicates that the basic cardiovascular responses do not significantly vary in accordance with the dual constitutional types of Ayurveda, it suggests that the increase in DBP recorded immediately after performing the isotonic exercise for five minutes varies significantly in relation to *Prakriti* groups, and this increase is significantly minimal among VK group in comparison to VP and PK groups.

## 6. Correction of Reference 3

*Present version:* B. Prasher, S. Negi, S. Aggarwal, A. K. Mandal, T. P. Sethi, and S. R. Deshmukh, “Whole genome expression and biochemical correlates of extreme constitutional types defined in Ayurveda,” *Journal of Translational Medicine*, vol. 6, article no. 48, 2008.

*To be corrected as:* B. Prasher, S. Negi, S. Aggarwal et al. “Whole genome expression and biochemical correlates of extreme constitutional types defined in Ayurveda,” *Journal of Translational Medicine*, vol. 6, article no. 48, 2008.

## 7. Correction of Reference 4

*Present version:* K. Patwardhan, Human Physiology in Ayurveda, Jiakrishnadas Series No.134, Chaukhambha Orientalia, Varanasi, India, 2008.

*To be corrected as:* K. Patwardhan, Human Physiology in Ayurveda, Jaikrishnadas Series No.134, Chaukhambha Orientalia, Varanasi, India, 2008.
